# Leading Change at Berkeley Public Health: Building the Anti-racist Community for Justice and Social Transformative Change

**DOI:** 10.5888/pcd20.220370

**Published:** 2023-06-08

**Authors:** Amani M. Allen, Ché Abram, Navya Pothamsetty, Andrea Jacobo, Leanna Lewis, Sai Ramya Maddali, Michelle Azurin, Emily Chow, Michael Sholinbeck, Abby Rincón, for the ARC4JSTC Action Team, Ann Keller, Michael Lu

**Affiliations:** 1 School of Public Health, University of California, Berkeley; 2 Rhodes College, Memphis, Tennessee; 3 University Library, University of California, Berkeley

## Abstract

A transformative change grounded in a commitment to antiracism and racial and health equity is underway at the University of California, Berkeley, School of Public Health. Responding to a confluence of national, state, and local circumstances, bold leadership, and a moral and disciplinary imperative to name and address racism as a root cause of health inequities, our community united around a common vision of becoming an antiracist institution. Berkeley Public Health has a long history of efforts supporting diversity, equity, inclusion, belonging, and justice. Building upon those efforts, we pursued an institution-wide initiative, one that creates a more equitable and inclusive school of public health that models and supports the development of future public health leaders, practitioners, scholars, and educators. Grounded in the principles of cultural humility, we recognized that our vision was a journey, not a destination. This article describes our efforts from June 2020 through June 2022 in developing and implementing ARC4JSTC (Anti-racist Community for Justice and Social Transformative Change), a comprehensive, multiyear antiracist change initiative encompassing faculty and workforce development, student experience, curriculum and pedagogy, community engagement outreach, and business processes. Our work is data informed, grounded in principles of change management, and focused on building internal capacity to promote long-term change. Our discussion of lessons learned and next steps helps to inform our ongoing work and antiracist institutional change efforts at other schools and programs of public health.

**Summary**

**What is already known on this topic?**

Schools and programs of public health (SPPH) have a moral, ethical, and disciplinary imperative to address problems that undermine our collective mission to improve health and well-being for all. Many SPPH have declared racism a public health crisis, but little guidance exists in the published literature for addressing racism, including structural racism, in academic public health.

**What is added by this report?**

We describe ARC4JSTC, an inclusive data-informed initiative at the University of California, Berkeley, School of Public Health, for actively working toward becoming an antiracist institution.

**What are the implications for public health practice?**

We conclude with a discussion of lessons learned and next steps to inform antiracist institutional change efforts in SPPH.

## Background

The permanence of racism as an enduring feature of society is well-documented (1,2). It is embedded in all institutional structures, including higher education (3). Academic public health has a moral, ethical, and disciplinary imperative to address problems that undermine our collective mission to improve health and well-being, particularly for oppressed groups who, due to the concentration of privilege, that is, “when one group has something of value that is denied to others simply because of the groups they belong to,” (4) have suffered the *dis*accumulation of protective resources and *hyper*accumulation of risk (3,5). Fulfilling that charge will require doubling down on our efforts to hold ourselves accountable including critical self-reflection as institutional change agents to ensure, internally, we are up to the task.

Discussions of racism and antiracism in higher education are not new but have resurged in recent years (6–20). Scholars and administrators alike have focused attention on the structural ways in which racism operates in higher education, noting that racism is multilevel and multifaceted and, thus, that interventions must also be multilevel and multifaceted (6,21,22).

The American Public Health Association, the Council on Education for Public Health, and the Association for Schools and Programs of Public Health have each declared racism a public health crisis and taken steps to promote antiracism in the profession and support schools and programs of public health (SPPH) with the tools for antiracist transformation (Table 1) (23–28). These efforts demonstrate a clear trend toward SPPH being intentional about transforming into diverse, equity-minded, inclusive, and antiracist institutions.

**Table 1:** Support of Antiracist Transformation in Schools and Programs of Public Health at Leading Public Health Organizations

Year	Organization	Action
2015–2016	American Public Health Association (APHA)	Then-APHA President Camara Jones launched a national campaign against racism as the primary agenda of her presidency, raising awareness of racism as a root cause of racial health disparities (23).
2016	Council for Education in Public Health (CEPH)	CEPH developed a new foundational competency to ensure that racism would be addressed in the master of public health curriculum at all accredited schools and program of public health and students would be equipped to face the challenges of effective public health practice (24): “Discuss the means by which structural bias, social inequities and racism undermine health and create challenges to achieving health equity at organizational, community and societal levels.”
2019	APHA	“Racism: Science & Tools for the Public Health Professional” was published by APHA (25), “designed to arm public health professionals with 1) knowledge about the relationship between racism and health; 2) tools to address racism; and 3) inspiration to pursue health equity.”
2020	Centers for Disease Control and Prevention	Launched an updated 10 Essential Public Health Services (EPHS), intended as a framework for achieving health equity by protecting and promoting the health of *all people in all communities* (26). EPHS “seeks to remove systemic and structural barriers that have resulted in health inequities. Such barriers include poverty, racism, gender discrimination, ableism, and other forms of oppression.”
2020	Association for Schools and Programs of Public Health (ASPPH)	Issued a statement of commitment to zero tolerance of harassment and discrimination in schools and programs of public health, including 5 tenets to help guide strategic action in SPPH (27): 1) antiharassment and anti-discrimination policies and trainings, 2) identifying and reporting harassment and discrimination, 3) protecting victims of harassment and discrimination, 4) communicating and transparency, and 5) shifting the culture.
2021	ASPPH	Issued a framework for dismantling racism and structural racism in academic public health, which includes a shared vision for academic public health as diverse, equitable, inclusive, and antiracist (28). They provide short, intermediate, and long-term goals as well as specific actions that SPPH can take toward fulfilling that charge.

Useful guidance for such efforts has been published by SPPH, other institutions of higher education, and health equity scholars (11,15,21,29–33). Despite this body of work, published evidence that documents or provides guidance for antiracist transformation within SPPH is scant. We help fill that gap by documenting our efforts at Berkeley Public Health (BPH). This article describes our process for leading antiracist change, discusses our successes and challenges, and provides recommendations for antiracist institutional change efforts at other SPPH.

## Berkeley Public Health: the Local Context

### Past successes and challenges

Social justice is a deeply rooted pillar of BPH, guiding our organizational mission, values, principles of community, and our ongoing diversity, equity, inclusion, and belonging (DEIB) efforts. Over the years, these efforts included hiring our first-ever director of diversity in 2005 which resulted, in part, from student advocacy and aligned with campuswide efforts to support DEIB at the local level (ie, schools and colleges) with demonstrated impacts in increasing underrepresented minorities in applicant pools and in matriculation; creating a diversity services office in 2005, which expanded into the Diversity, Respect, Equity, Action, Multiculturalism (DREAM) Office in 2015 with an explicit focus on inclusion and belonging, intensified efforts to increase diversity of the student body (recruitment, retention, and graduation), and consultation regarding admissions policies and improving faculty diversity; student-led town halls on racism, power, and privilege in 2015; creating the Diversity, Inclusion, Community, Equity Committee in 2015 — a voluntary collective of students, staff, faculty, and alumni addressing equity issues at BPH — which was the result of student activism and an outgrowth of the student-led town halls; conducting surveys to monitor BPH climate starting in 2015; developing new curricular competencies that address structural inequities in 2016 (eg, structural competence — critical thinking about real-time issues of structural competency, health inequity, and antiracism in public health practice and research); adding course evaluation questions on classroom climate and respectful student engagement starting in 2018; training faculty and staff on having courageous conversations about race, understanding and addressing racial microaggressions, and bystanderism (not intervening despite witnessing or being aware of a racist act) in 2019; and hiring a full-time Dean’s Cabinet–level chief of DEIB (2021). These are tremendous successes. However, like many schools, we have faced challenges: for example, continued reports of microaggressions and other experiences that hinder inclusion, sense of belonging, and schoolwide ownership of DEIB.

### A call to action

On June 9, 2020, Dean Michael Lu issued a statement condemning racism and all forms of White supremacy and declaring racism a significant determinant of health. The statement — drafted in collaboration with several faculty and staff, including those from underrepresented racial and ethnic minority groups — drew broad support across BPH constituencies and catalyzed the groundswell for creating a more equitable and inclusive school of public health. The resulting schoolwide advocacy for intensifying our institutional commitment to antiracist change aligned with the efforts of a large group of faculty, students, and staff already working on antiracist efforts. The “Solidarity Against Racism During Covid-19” group organized because of racist hate emails received by a faculty member who spoke nationally about the disproportionate harms of COVID-19 among racial and ethnic minorities. The group issued a call to action and organized around several efforts, including a collaboration between faculty and students to develop a BPH website highlighting evidence that documented the effects of structural racism on health. The objective of the website was to ensure that the BPH community itself understood these fundamentals and could turn to this resource in their own research, teaching, mentoring, and community and professional service.

In a follow-up to his June 9th statement and against a backdrop of already lively antiracism activities, Dean Lu created 4 workgroups: 1) building an antiracist curriculum; 2) antiracism and racial justice training for faculty and staff; 3) expansion of supports for underrepresented students, faculty, and staff; and 4) collaboration between finance and development to identify existing funds and generate additional resources to sustain BPH antiracism efforts. Each group was cochaired by a member of the Dean’s Cabinet to ensure institution-level engagement and accountability (Table 2).

**Table 2:** Initial Charge for Antiracism Project Teams at the University of California, Berkeley, School of Public Health

Project team	Initial charge	Accountability team
Antiracist curriculum	Expand antiracist training (including addressing racism as a public health issue) throughout our curriculum; review our curriculum and identify opportunities to strengthen antiracism training throughout.	Executive associate dean, chief of curriculum and instruction, Education Policy and Curriculum Committee
Faculty and staff training	Mandatory implicit bias and antiracist training for all faculty and staff, similar to a voluntary “Beyond Diversity” training offered in 2019.	Chief operating officer and Diversity, Inclusion, Community, Equity (DICE) Committee
Recruitment and supports	Identify opportunities to expand outreach, recruitment, supports, networking, and mentoring for underrepresented minority students.	Assistant dean of students, interim chief of DEIB and the Diversity, Respect, Equity, Action, Multiculturalism (DREAM) Office
Diversity, equity, and inclusion (DEI) support	Develop plans for strengthening DEIB for underrepresented staff and faculty.	Dean, Faculty Council, Staff Advisory Council
Resources	Identify existing funds and generate additional resources to support and sustain Berkeley Public Health antiracism efforts; without additional resources and support, these efforts often fall on the DREAM Office, the DICE Committee and underrepresented faculty, staff, and students and are not sustainable.	Finance team and development team

To ensure action at multiple levels, Dean Lu asked each division (eg, epidemiology, environmental health sciences) to consider what actions they might take to support the school’s antiracism goals. We define racism as a *system* of structuring opportunity that confers unfair advantage and disadvantage by race across multiple levels, from structural and institutional policies, practices, and norms, including (control over) collective and individual discourses (34) — systems of thoughts, constructed knowledge, beliefs, attitudes, and communications that construct or govern interpretations of reality/truth — to individual beliefs, attitudes, and behavior. We further acknowledge that racism intersects with other forms of oppression to create unique intersectional risks and harms (35).

We faced a unique window of opportunity, and because of a confluence of factors, we were ready to meet the moment. One major factor was our new dean. Less than a year into his deanship, at a BPH faculty meeting in November 2019, Michael Lu named social inequality as one of the “most pressing issues of our time.” He made it a focus of his leadership at BPH and 1 of 4 priority areas for the school. With the right leadership, the strong national, local, and disciplinary imperative to address structural racism as a public health issue, and an already activated community, we were primed for change.

## ARC4JSTC: Planting the Seeds of Change

### Phase 1: Coalition building

The initial burst of activities (summer 2020) was relatively uncoordinated; action was disaggregated at multiple levels, which risked inefficiency (eg, wasted human, time, and other resources) and burnout, impeding co-learning, and ultimately undermining long-term success. To address this concern, our executive associate dean (A.M.A.), also chair of ARC4JSTC, convened the cochairs of each workgroup, a division chair representative, several student representatives, the school’s equity advisor, and the chief of curriculum and instruction to discuss a coordinated effort. This initial group grew organically, adding voices and perspectives that were missing from early discussions. We were deliberate about creating a steering committee that was as inclusive as possible while avoiding overlap to ensure efficiency. For example, it was essential to have a representative cross-section of various divisions (including the joint medical program), offices, committees, and operational roles; student (undergraduate and graduate), staff, faculty, and alumni representatives; registered student organizations; and racial and ethnic diversity as well as diversity in other forms of social identity; community representation; and dean’s office representatives to ensure continued institutional commitment. Ex-officio members were also available to ensure alignment with relevant campus initiatives and support co-learning with other campus units; these individuals had operational roles needed for successful implementation (eg, BPH assistant dean of finance, BPH director of education operations, campuswide director of DEIB). After 6 months of sharing insights from different perspectives about organizational goals, strengths, challenges, opportunities, and potential threats, we had a 23-member steering committee that represented the knowledge and perspectives, skill sets, and operational areas integral to seeding change. We asked each committee member the following questions: 1) What future do you imagine for an antiracist BPH? 2) What do you believe is most important for us to keep in mind with this work moving forward? and 3) What will help you stay committed to this work moving forward? Responses were integrated into a set of guiding principles (Box).

**Box. ARC4JSTC (Antiracist Community for Justice and Social Transformative Change) Action Team Guiding Principles, University of California, Berkeley, School of Public Health**

1. Striving for a new identity as an antiracist institution — identified both internally and externally — is an ongoing journey, not a destination.

2. Leading for antiracist change means full integration and normalization of antiracist praxis.

3. Racism is structural — deeply entrenched into organizational policies, practices, and norms — therefore, antiracist efforts must also be structural.

4. Identifying and remediating institutional racism is a marathon, not a sprint; it requires educating (learning and unlearning), organizing, nurturing, and holding each other accountable for meaningful change.

5. Knowledge of racism, power, and privilege; deep understanding of marginalizing experiences; and both individual and collective action are all critical to antiracist transformation and are the foundation for developing an equity and social justice–oriented praxis.

6. The process of antiracist change will challenge deeply held beliefs about power and privilege and require confronting resistance at the individual, interpersonal, and institutional levels.

7. Perseverance will require acknowledging and celebrating progress along the way.

8. Antiracist praxis will be new for some and will sharpen growth edges for others; and will require balancing accountability, empathy, and compassion among all.

9. The antiracist changes we make today are an investment in our future identity (see first guiding principle).

### Phase 2: Creating a vision and strategy for change

Recognizing the need for project management, we enlisted the support of our campus Business Process Management Office. Their investment in supporting the university’s goal of becoming an antiracist campus created a mutually beneficial partnership. We received 3 forms of support:

1. Project management — managing and organizing work, meeting timelines, coaching project sponsor and project chair, and tracking progress
2. Change management — creating a management structure to demonstrate the organization’s commitment to change and creating a resistance management plan including coaching project leaders on communications and managing internal relationships
3. Survey design and testing — optimizing user experience via item formatting and ordering and managing alpha and beta testing.

#### Data collection

Given the need to align our strategies with the needs of our community (21,36), we collected various forms of data during summer and early fall 2020. Initial data collection included literature reviews on various topics: antiracism and antiracist praxis (19,21,31), antiracist and culturally responsive pedagogy (20,25), antiracism in institutions and in higher education (11,32), and examples of frameworks for antiracist change in organizations (28–30,36), including higher education (10,31,32). We conducted focus groups with faculty in each division and the joint medical program to assess their readiness for change and perceived challenges and opportunities for successful implementation. The executive associate dean and chief of curriculum and instruction used a semistructured focus group guide to facilitate faculty focus groups via Zoom. We conducted 7 focus groups, 1 for each of the 6 divisions and the joint medical program. Focus groups had 4 to 8 participants each. The participation rate was low (23%; 5 of 22) for the largest division because of scheduling challenges; participation ranged from 50% (4 of 8) to 89% (8 of 9) for other divisions. Faculty members used fictitious Zoom names to protect confidentiality, and transcripts were further de-identified by using letters or numbers to designate participants. Two former BPH students conducted transcription and thematic analysis. Student input was solicited via informal gatherings sponsored by the DREAM Office, the assistant dean of students, and the interim chief of DEIB. We also administered structured surveys to students, faculty, and staff and nonfaculty academics (eg, researchers, project scientists). Our schoolwide surveys assessed various factors, including racial literacy; bystanderism; motivation, readiness, confidence, and current practices related to antiracist pedagogy (in and outside the classroom); willingness to commit time to antiracist learning and the most desirable types of learning experiences; and perceptions of racial equity across organizational categories (eg, hiring and retention). Survey data collection was successful, particularly among our faculty (89% response rate; 141 of 159). We received 231 completed student surveys (21% response rate; 231 of 1,111), and 74 staff and nonfaculty academics completed surveys (69% response rate; 74 of 107). We also reviewed the past 5 years of BPH climate survey data to generate a snapshot of school climate. We used results from all data collection to inform program planning.

#### Project charters

Each workgroup then created a project charter that outlined short-, intermediate-, and long-term goals, objectives, strategies, and metrics for evaluating success. Informed by our data collection (ie, internal community needs assessment) and steering committee and project management discussions, our project structure changed from 4 workgroups (Table 2) to 4 population-specific project teams to ensure that the holistic needs of each constituency were considered: faculty development, curriculum, and pedagogy; student experience; workforce development; and community engagement. For example, for students, in addition to outreach, recruitment, networking, and mentoring, we examined other supports students may need and want to promote DEIB and ensure that students were thriving. For faculty, we considered supports needed to expand antiracist training throughout the BPH curriculum. We also developed a set of cross-cutting foundational teams whose work addressed core business practices and was critical to overall program success:

1. Business process and practice — faculty and staff recruitment policies and practices, purchasing practices
2. Data and evaluation — collection, analysis, and dissemination of data to support planning and evaluation efforts
3. Voice and visibility — improvement in understanding of the health consequences of racism and communicating the antiracism work being done in the school for all BPH community members
4. Change management – creation of an environment in which project management and change management converge to achieve organizational objectives.

#### Communicating the change vision

Committee members raised concerns about the term “steering committee” being hierarchical and antithetical to our goals of creating a more inclusive community. To foster community ownership of the work, we invited everyone in the school to provide recommendations for renaming the steering committee. We received 150 responses and found consensus on several terms and phrases, which resulted in a new name for the program and the steering committee: the Anti-racist Community for Justice and Social Transformative Change (ARC4JSTC) and the ARC4JSTC-Action Team (ARC4JSTC-AT), respectively.

In March 2021, the ARC4JSTC-AT conducted a listening tour to communicate and solicit feedback on the change vision and plan throughout the school. Feedback was overwhelmingly positive. In the interest of developing short-term wins to motivate continued engagement and growth, we prioritized activities that 1) would build internal capacity, 2) could be implemented quickly and with current resources, 3) would have high visibility (ie, to convey institutional commitment), and 4) would have recognizable and sustainable impact. After finalizing the plan, the ARC4JSTC-AT provided the dean with a budget request, met with campus groups and offices to communicate our plan for antiracist transformation, and secured additional funds and other in-kind support (ie, office of the executive vice chancellor and provost, People & Culture [staff services], private donors, a foundation grant, and a faculty climate pilot grant from the University of California Office of the President).

### Phase 3: Project implementation

Data collection revealed several growth areas for the BPH community. The most obvious were racial literacy, bystanderism, and the skills and confidence to implement antiracist praxis. Previous climate survey data showed that although most survey respondents indicated experiencing BPH as welcoming (91%; 316 of 347), respectful (86%; 300 of 347), supportive (80%; 279 of 347), inclusive (78%; 272 of 347), and diverse (71%; 248 of 347), more than one-quarter of respondents reported perceived and experienced racial and other forms of bias and discrimination, most commonly caused by faculty.

Each project team undertook multiple projects (Table 3). Following is a description of 4 projects, one for each project team.

**Table 3:** Implementation Plan for Antiracist Community for Justice and Social Transformative Change (ARC4JSTC), University of California, Berkeley, School of Public Health (BPH)

Strategy	Project status
**Foundational and baseline antiracism and racial equity trainings for faculty (mandatory)**: Develop foundational racial literacy for all BPH faculty. Includes discussion of White supremacy, axes of power and privilege, racial identity, stigma and implicit bias, and experiential skills-building in facilitating tense classroom conversations.	Completed
**Antiracism and racial equity workshops for new students**: Incorporate antiracist praxis training as part of new student orientation.	Ongoing (yearly)
**Elective series of antiracism trainings and community building for staff and nonfaculty academics**. 1) Elevation 2 Transformation (fall 2021): 1-day virtual seminar intended to develop a foundation and provide tools for talking about race both interracially and intraracially. Includes exercises to elevate racial consciousness, develop a deeper understanding of the impact of race, and gain clarity around the construct of Whiteness and its role in sustaining systemic racism; 2) Deeper Dive (fall 2021): An advanced 3-part series open to those who had completed 1 of 2 prior racial equity trainings (Courageous Conversations or Elevation 2 Transformation). Participants focus on deepening understanding of race and systemic racism by examining their organization/department’s policies, practices, programs, structures, climate, and culture through an ongoing cycle of inquiry; 3) Leader of Leaders (spring 2022): A 4-part series where participants learn to recognize destructive patterns that maintain the status quo and gain the skills to create solutions and disrupt inequities personally, professionally, and organizationally.	Completed
**Antiracist and Racial Justice Praxis graduate student elective**: Cultivate student champions to develop an antiracist analysis of public health, present a set of antiracist public health tools, and build skills necessary for advancing an antiracist agenda within the field.	Ongoing (yearly)
**Antiracist Pedagogy Faculty Leadership Academy**: Develop a cohort of antiracist faculty champions/early adopters that will lead in curricular transformation (integrating antiracism and racial equity competencies into core curriculum and BPH leadership experiences) and serve as coaches/trainers for other BPH faculty.	Completed
**Antiracist/racial equity community agreements on all BPH syllabi**: Collaborate with Faculty Council to pass a resolution requiring a clear statement about commitment to antiracist and racial equity teaching practices. Includes language sample and resource guide.	Completed
**Schoolwide antiracist and racial equity competencies**: Develop a set of schoolwide and group-specific antiracist competencies to be implemented and operationalized schoolwide.	Implementation phase
**Bias reporting form**: Monitor incidents of bias (many of which currently go unreported based on climate survey data) and document positive examples of antiracist praxis to disrupt continued mistreatment, use as a resource for promoting effective antiracist praxis, and inform our ongoing antiracist efforts.	Implementation phase
**Antiracism website**: Make a public commitment to antiracism and track our progress and processes, including an entire section that provides scholarship on how racism impacts health.	Completed
**Planning for a BPH Community Advisory Board,** including funded positions for community advisors to support strategic planning efforts.	Ongoing
**Antiracist procurement**: BPH commitment to prioritize minority-owned business vendors and more generally promote practices within BPH and across campus using a DEIBJ (Diversity, Equity, Inclusion, Belonging, and Justice) lens for procurement activities.	Ongoing
**Antiracist staff hiring protocols**: Introduce DEIBJ assessment into the staff hiring process.	Completed
**Create a standardized faculty search plan** that incorporates DEIBJ best practices and is consistent with our goals to diversify the faculty (collaboration between administration, faculty, and students).	Completed
**Strategic planning for integrating antiracist pedagogy throughout BPH**: Develop DEIBJ goals, objectives, strategies, and metrics for evaluating short, intermediate, and long-term success; incorporate ARC4JSTC as central pillar.	Implementation phase
**Identify existing human capital and curricular strengths/assets and limitations/liabilities to guide ongoing curricular planning and pedagogic transformation.**	Planning phase
**Establish and implement plan for ongoing antiracist/racial equity training for BPH faculty, staff/nonfaculty academics, and students.**	Planning phase
**West Coast Public Health Anti-racism Collaborative (WPH-ARC)**. Develop a collective of West Coast schools of public health actively engaged in antiracism efforts as a source of support for those engaged in this work and to identify opportunities for collaborative efforts to scale the impact of our individual efforts. Engaged schools: BPH; Portland State University; University of California, Los Angeles; University of California, San Diego; University of Washington.	Implementation phase

#### Antiracist Pedagogy Faculty Leadership Academy

In late spring and early summer 2021, BPH implemented its inaugural Antiracist Pedagogy Faculty Leadership Academy. The Academy was designed to follow up on an initial set of mandatory introductory racial literacy faculty workshops (100% participation, completed in fall 2020) for faculty interested in further developing their antiracist praxis and to create an early adopter group of antiracist champions to support ongoing curricular transformation. Part I (early summer) consists of five 2-hour didactic and active learning sessions focused on applying historical and contemporary, conceptual, and practical lenses to the subject of racism, antiracism, and antiracist pedagogy while developing an opportunity for collaborative learning and strategizing. Participants learn foundational theories and frameworks for understanding structural racism, particularly in higher education; connect this scholarship to their teaching through reflection and discussion with colleagues; create and adapt strategies to redesign their syllabus; and develop and practice pedagogic skills that foster inclusive classroom environments. Part II, “Implementing Your Antiracist Curriculum,” consists of monthly Community of Practice Learning Laboratories during the fall and spring semesters. These sessions provide an opportunity for faculty to discuss their overall classroom environment, including events occurring in the classroom related to DEIB and racism and antiracism more generally, troubleshoot, and continue to work on developing antiracist pedagogy skills.

For our inaugural Academy, we invited selected faculty members (N = 39) to maximize the number of students impacted and ensure faculty training for a cross-section of our programs and divisions. This included faculty from our core and breadth classes, leadership classes, interdisciplinary programs (doctor of public health core seminar, interdisciplinary master of public health core seminar, online master of public health classes, joint medical program), and graduate student instructor pedagogy course. We conducted surveys after each session to assess the effectiveness of the material and the instructor and a presurvey and postsurvey to assess the effectiveness of the Academy in supporting the development of antiracist pedagogy skills. Responses indicated that the Academy helped improve participants’ perceived knowledge, skills, readiness, and confidence in a range of antiracist pedagogy practices. We also held a post-Academy listening session via Zoom and received helpful feedback for session logistics and for modifying our evaluation strategy, including the frequency of surveys. Participants indicated that completing a survey after each session was burdensome. Participant feedback was positive overall:

- “This training was essential and foundational.”
- “Gave me space to be so much braver in my classes. It was almost like magic. This semester has been one of my most fulfilling semesters of teaching.”

#### Antiracist and racial justice praxis graduate student elective

A new graduate student elective course teaches students how to develop an antiracist analysis of public health, present a set of antiracist public health tools, and build skills necessary for advancing an antiracist agenda in the field. The course consists of 4 competencies and multiple learning objectives (Table 4) and was approved by our Education Policy and Curriculum Committee. The course was initially offered in spring 2021 and is now offered each spring semester given the highly favorable ratings for instructor effectiveness, course effectiveness, and classroom climate: 6.9, 6.7, and 6.6 on a scale of 1 (low) to 7 (high), respectively.

**Table 4:** Antiracist and Racial Justice Praxis Graduate Course, University of California, Berkeley, School of Public Health (BPH)

Competency	Sample learning objectives
Distinguish the unique impact and history of White supremacy from other forms of oppression in the US, recognize how racism affects individuals and the field of public health, and analyze racial health disparities within the context of historical and current racism.	Understand the history of Whiteness and racism in the US and apply historical perspective when analyzing present-day racial challenges.
	Recognize the emotional impact of racism on behavior and develop new tools for emotional awareness and self-regulation.
	Analyze the role of racism and White supremacy in public health practice, programming, and research.
Apply antiracism principles to public health interventions, design new public health programs that address racism as a root cause, and modify existing programs to be more effective is addressing racism as an underlying cause of health inequities.	Describe the 4 components of an intervention that addresses racism as a root cause.
	Apply the racism-as-a-root-cause approach to develop antiracist programs and organizational strategic plans.
	Apply design thinking approach to develop antiracist interventions.
Cultivate transformative antiracist change by effectively engaging and empowering communities most impacted by racism, identifying institutional and legislative policies that will have an antiracist impact, and leveraging media and public communications tools to advance policy change.	Recognize the role of policy in antiracist change and understand how to use institutional and legislative policy to advance racial justice.
	Understand what effective community engagement and power-sharing is and describe key strategies to ensure high-quality community engagement.
	Learn how to leverage news media to create political pressure and advance political change.
Formally evaluate the racial impact of research and public health interventions, refine existing programs to integrate antiracist strategies, modify mainstream quality improvement tools so that they can measure antiracist impact, and sustain ongoing antiracism change within and beyond the field of public health.	Apply the racism-as-a-root-cause approach and racial equity impact assessment tools to assess racial impact of existing research programs and public health interventions.
	Leverage quality improvement tools to improve the antiracist impact of existing research programs and public health interventions.
	Apply communication skills to engage in effective racial dialogue.
	Assess personal positionality and associated risk in advancing antiracist organizational change.

#### Antiracist training and community building for staff and nonfaculty academics

Our workforce development team created a work plan based on a series of planning and brainstorming sessions during the 2020–2021 academic year. As a result, a series of voluntary trainings and community building circles was held in collaboration with an outside vendor (race-work.com) and our campuswide Restorative Justice Center (Table 3). Community building circles were focused on setting the stage for some of the content that would be covered in the training. The circles helped us build trust, establish community agreements, and start to develop tools and skills for building empathy and responding to conflict in positive and transformative ways (36). We conducted a survey in December 2021 to solicit feedback from attendees. In addition to other questions, respondents were asked to assess their capabilities on a set of antiracist practices after the trainings, compared with before. Respondents reported feeling more motivated, ready, and confident to participate in antiracist practices after the fall 2021 trainings: 72%, 76% and 63%, respectively. Responses also indicated a strong motivation to make BPH more antiracist and the important role of community and trust building for enabling a sense of personal and collective responsibility. Results for spring 2022 were similar. Trainings have continued each semester, and additional planning is underway.

#### Community Advisory Board planning

The goal of the community engagement project team is to help ensure community voices are represented in BPH’s decision making and efforts to become an antiracist institution. The initial project was to develop plans for a schoolwide community advisory board and to reimagine what community engagement can or should be. To ensure community voice during the planning process, we recruited 5 community members through an open call for applications to serve on the planning team, each receiving a $3,000 stipend for their participation. Planning is ongoing. Next steps are to ensure alignment with the vision of school leadership and other constituency groups (eg, students, faculty).

### Empowering the BPH community for long-term change: shifting the culture

Three major projects are underway. The first is to develop a set of antiracist competencies, or habits of heart and mind (37) — instinctive ways of being and thinking — that we aspire to and that will help inform our ongoing planning. These competencies are intended to characterize how we want to “show up” as a school in relation to antiracism, racial equity, and equity more broadly. The foundational work has been completed — literature reviews, focus groups, and interviews — and a draft set of 3 competencies was developed and vetted among BPH groups. They are racial literacy, cultural humility, and collective responsibility. Our next step is developing a plan to operationalize them. For example, how might these competencies be operationalized to further inform our educational competencies and curriculum? How might they inform our pedagogic practice and course learning objectives? How might they inform continued faculty and staff development individually and collectively? The greatest impediment was pushback from some about the term “accountability,” which was ultimately changed to “collective responsibility” to avoid a stalemate; although many students, faculty, staff, and the ARC4JSTC-AT felt “collective responsibility,” although important, deflected personal responsibility.

Second, we are in the final stages of developing a bias reporting form. The form will provide an opportunity for anonymous reporting of bias and discrimination of any sort and will also be designed to capture examples of positive experiences. Our goals are to 1) monitor our progress (we expect the number of reports to decline over time as we become more antiracist) and 2) develop a library of cases that we can use as a resource for future trainings. Third, we are also in the final stages of seeking feedback on our new schoolwide DEIB plan. The plan includes a set of goals, objectives, and current and future strategies in 4 focus areas: teaching and learning, social impact, belonging, and infrastructure. Next steps are to develop a set of metrics to evaluate success. The plan rests on 2 pillars — antiracism and social justice — and aims to advance our efforts toward becoming an antiracist institution.

## Lessons Learned and Recommendations

Our formal data collection activities and informal feedback from individual and group discussions and presentations of our work informed our understanding of some of the challenges of antiracist change. First is the importance of a resistance management plan. Antiracism is not a universally accepted concept (38) and, as discussed by the West Coast Public Health Antiracism Collaborative in biweekly meetings during 2021 (Table 3), the existence of structural and other forms of racism at SPPH is also not universally accepted. Hence, resistance is inevitable. Although there is no single approach to *doing* antiracism, there exists deep and well-tested knowledge on a plethora of practices for creating antiracist institutional change. Racial literacy (39) is paramount. Bringing in experts to achieve common understanding and start to build individual and collective critical consciousness is essential. Not doing so opens the door for misinformation and misunderstandings, which complicates achieving a unifying vision for any type of antiracist work. Furthermore, commitment to broad-scale change increases when motivation is intrinsic (40). Hence, the process of learning and *un*learning is critical to antiracist change efforts (21). Establishing a common understanding and common vision is also an essential component of establishing a resistance management plan. Establishing norms for open and honest communication is critical. Uplifting the lived experience of racial and ethnic minority populations as a source of information for understanding *how* racism operates is a necessary component of achieving shared understanding. White people will need to be receptive and respectful of the lived experience of their non-White colleagues (peers, students, teachers, administration). This is a question of epistemology: whose knowledge is valued and considered as valid data to help understand the phenomenon of interest? Experiential knowledge is a central tenet of critical race theory, as is understanding that an individual need not be overtly prejudiced or commit acts of prejudice for racism to flourish (31). Beverly Tatum’s image of the moving walkway is illustrative (41). One need not be actively racist to promote or endorse racism (a system). Simply standing on the moving walkway and being a recipient of unearned privilege while others are on different walkways entirely is an endorsement of the status quo. Similarly, Jones’ articulation of acts of commission and acts of omission illustrates the passive endorsement of status quo structural racism (42). Thus, it is essential to understand how racism is operating within the local context to inform targeted strategies (21,36).

Feelings of fear and guilt among White people and perceived hostility toward White people can create conflict and are well-known barriers in antiracist change efforts (36). Emotions are intrinsic to antiracist change. For historically marginalized groups, antiracist change is long overdue, whereas for many White people, it challenges an image of the self as liberal and caring and of the status quo as being neutral. It is also fraught with concerns about getting it right for fear of discomfort in and outside the classroom and of blame as one starts the bold *process* of change. Thus, psychological *un*safety is a necessary part of antiracist transformation (16,17,20,43), an inherently disruptive process (18).

Allies are also critical to resistance management and for building bridges and challenging the status quo (21). For example, it took White allies to speak up against the notion that antiracist praxis is not possible in methods classes for our resolution to include a statement of commitment to antiracist pedagogy on all BPH syllabi to be approved unanimously (Table 3). Identifying points of convergence and divergence in understanding will be key to having courageous conversations about race, which is essential for doing the hard work of exposing, confronting, and combatting racism. Having a solid evaluation plan up front is also an important aspect of resistance management. We found people eager to participate in trainings but reluctant to be held to account. Thus, an inclusive process to establish evaluation strategies and getting early buy-in is essential. However, recognizing points of conflict and continuing to pursue goals even in light of conflicting views will also be part of the process.

Second, institutional commitment and long-term investment is essential (21,36). Administrative leadership helps establish an initial sense of urgency, plays an important convening role in inducing enthusiasm for getting involved, articulates alignment with organizational goals, and demonstrates a high level of commitment to overall program success (44). Although we were successful in securing funds outside of BPH, a stable budget from BPH to underwrite the work was critical to planning. Additionally, having a multiyear budget also helped support long-range planning efforts. Although financial investment is important, a more comprehensive perspective in determining how various institutional resources (eg, financial, human) can be used to support program planning, implementation, and sustainability will ensure proper infrastructure to support the work. It is essential that those commitments be communicated broadly. Frequent communication to the broader school community also demonstrates institutional commitment and can become a vehicle for soliciting feedback and participation and helps create buy-in.

Third, ARC4JSTC-AT members expressed concerns about burnout as a result of taking on additional time, labor, and emotionally taxing activities while maintaining their regular scope of work. This resulted in a conflict between their desire to remain engaged and complete tasks in a timely manner and their capacity for taking on the additional workload. This was particularly important for staff and students, for whom “service” was not part of their regular responsibilities. For racial and ethnic minority faculty members, particularly those in predominantly White institutions, a strong body of work documents statistically significant associations of reported racial discrimination, vocational strain, and role overload with mental and physical health and well-being, research productivity, work strain, and an overall unwelcoming climate. Studies document substantial emotional labor among underrepresented minority faculty at predominantly White institutions due to the disproportionate burden of formal and often informal and invisible service (eg, student mentoring, peer mentoring) (45); and the disproportionate burden of DEIB work, particularly in predominantly White institutions, where racial microaggressions and other displays of racial bias and discrimination are rampant, making that work even more draining and distracting (46). Staff members who are members of racial or ethnic minority groups and other marginalized identities also experience the tax of providing disproportionate informal support to underrepresented minority and other marginalized students (47). Team members requested compensation for the additional workload or removal of other responsibilities to make room for the additional work effort. Considering how people will be compensated or otherwise credited for the work, or other strategies to reduce burnout, promote morale, and provide other supports is essential to maintaining the needed workforce and further demonstrating institutional commitment and appreciation of those involved.

## Conclusions

Antiracism praxis uses a structural approach to identify and address how racism operates within systems, going above the level of the individual to address change at the institutional level (21). Both a process and an outcome (29), racism operates in higher education through policies, procedures, curriculum and pedagogy, hiring, retention, promotion, admissions, resource allocation, climate, and culture, producing outcomes that maintain historical patterns of inequities (8,9). SPPH have a moral, ethical, and disciplinary imperative to support training, research, and service activities that serve our collective mission to promote health and well-being for all. Ensuring our institutional health as a diverse, equity-minded, inclusive, and antiracist-striving organization is fundamental to those efforts. We described our process of developing an ARC4JSTC, discussed successes and challenges, and provided recommendations for antiracist transformation at other SPPH. Change management, project management, a strong guiding coalition, and engaged commitment from institutional leaders helped provide stability through our change process and have been essential to sustained action.
